# Agar Hydrogel Template Synthesis of Mn_3_O_4_ Nanoparticles through an Ion Diffusion Method Controlled by Ion Exchange Membrane and Electrochemical Performance

**DOI:** 10.3390/nano9040503

**Published:** 2019-04-01

**Authors:** Qian Xue, Qiang Zhang

**Affiliations:** School of Chemistry and Chemical Engineering, Beijing Institute of Technology, 5 Zhongguancun Street, Haidian District, Beijing 100081, China; xueqian1070@163.com

**Keywords:** ion diffusion, ion exchange membrane, Mn_3_O_4_ nanoparticles, agar hydrogel, electrochemical performance

## Abstract

A novel strategy, ion diffusion method controlled by ion exchange membrane combining with agar hydrogel template, was reported for the synthesis of Mn_3_O_4_ nanoparticles without any oxidizing agents. X-ray diffraction (XRD), scanning electron microscopy (SEM), transmission electron microscopy (TEM), X-ray photoelectron spectroscopy (XPS) and Brunauere-Emmette-Teller (BET) isotherm were carried out to characterize the structure, morphology, pore size and distribution and specific surface area of the as-prepared nanomaterials. It is shown that the morphology and size of Mn_3_O_4_ nanoparticles can be controlled by the concentration of agar hydrogel. All the specific capacitances of the Mn_3_O_4_ samples prepared with agar hydrogel template are much higher than that of Mn_3_O_4_ prepared without any template agent. The Mn_3_O_4_ sample prepared at 1.5 g L^−1^ of agar hydrogel solution exhibits a highest specific capacitance of 183.0 F g^−1^ at the current density of 0.5 A g^−1^, which is increased by 293% compared with that of Mn_3_O_4_ synthesized without any template agent. The results indicate that the ion diffusion method controlled by ion exchange membrane combining with agar hydrogel template is a convenient and effective approach for preparing inorganic nanomaterials.

## 1. Introduction

As a new type of energy storage device, supercapacitors have the advantages of long cycle life, high power density, high safety and environmental friendliness [1,2,3,4], and have been applied in many fields [5]. According to their energy storage mechanism, supercapacitors can be classified as pseudocapacitors, electrical double layer capacitors (EDLCs) and hybrid supercapacitors [6]. Pseudocapacitors can achieve energy storage and release through highly reversible adsorption/desorption processes or fast redox reactions of the electrode active materials in the electrolyte. As the electrochemical reactions of pseudocapacitors occur both on the surface and inside of electrode, the energy density and specific capacitance of pseudocapacitors are usually much higher than that of other electrochemical capacitors [7,8].

Electrode active materials largely determine the performance of supercapacitors [9], and the morphology and structure of electrode materials have a great influence on the behavior of pseudocapacitor [10]. The reported pseudocapacitive materials mainly include transition metal hydroxides and oxides [11,12], carbon based electrode materials and conducting polymers [13] such as RuO_2_ [14], MnO*_x_* (*x* = 2 or 3/4) [15] and polythiophene derivatives (PTh) [16]. Transition metal oxides undergo rapid reversible redox reaction and exhibit an excellent pseudocapacitive performance. However, some of the transition metal oxides, for example RuO_2_, are rare and expensive, so they are not widely used in commercial application [2]. Therefore, Manganese oxide has become the suitable substitute of noble metal oxides and a popular research topic in recent years due to its abundant resources, environmental benignity, high theoretical specific capacity and capacitance retention [17,18].

Among the manganese oxide, MnO_2_ and Mn_3_O_4_ are two manganese oxides used as electrodes for supercapacitor. The spinel structured Mn_3_O_4_ are thermodynamically stable, which could avoid the structural collapse caused by proton intercalation and deintercalation in the electrode reaction process, showing excellent cycling stability and capacity retention [19]. The fact that Mn_3_O_4_ has only a single and stable hausmannite structure at room temperature makes the preparation of phase-pure Mn_3_O_4_ nanocrystals relatively easy. All these factors are advantageous to Mn_3_O_4_ as electrode materials for supercapacitor. Various methods such as hydrothermal method, solvothermal method and chemical bath deposition etc. have been successfully used to prepare Mn_3_O_4_ nanomaterials [20,21,22]. However, Mn_3_O_4_ has the disadvantages of low specific surface area, poor electronic conductivity, easy agglomeration in the preparation process and thus limiting its practical application. Various approaches have been developed to enhance its electrochemical performance such as reducing the particle size, and forming mesoporous structure [23,24]. Zhang et al. [25] have successfully prepared various Mn_3_O_4_ nanorods with different microstructures and nanostructures, and the Mn_3_O_4_ electrode has a specific capacitance of 136.5 F g^−1^ at a current density of 0.1 A g^−1^. Liu et al. have synthesized Mn_3_O_4_ solid nanospheres with a specific capacitance of 150 F g^−1^ at a current density of 0.3 A g^−1^ in 1 M Na_2_SO_4_ [26]. In our previous work [27], we doped Co ions in the Mn_3_O_4_ to prepare the Co(OH)_2_/Mn_3_O_4_ nanocomposites via a facile ion diffusion method, which effectively improved the aggregation behavior of Mn_3_O_4_ nanoparticles and increased the conductivity and specific capacitance.

The template method for preparing nanomaterials has the advantage of high repetition rate, controllable morphology, structure and size of the synthetic materials. It is classified as hard template method and soft template method [28]. In recent years, hydrogel has become an ideal template for preparing nanomaterials [29]. Hydrogel has a sponge-like three-dimensional network structure composed of polymer network and solvent. The unique three-dimensional network structure of hydrogel can provide the space for the nucleation and growth of inorganic nanoparticles, and the sizes of nanomaterials can be adjusted by modulating the pore size of the three-dimensional network of the polymer hydrogel [30,31]. At present, most of the hydrogel templates used for preparing nanomaterials are crosslinked polyacrylamide compounds, and inorganic salts solution is generally used as reactant and medium. The inorganic nanoparticles/hydrogel complex is obtained by the free radical polymerization and crosslinking reaction of acrylamide in the existence of crosslinking agent, and then the wet gel is dried and calcined to get inorganic nanomaterials [32,33,34]. This method takes the advantages of solid phase method and sol-gel method, which can mix various reactant ions uniformly at atomic level in aqueous solution, does not require the expensive alkoxides as reactants, and can control well the stoichiometric ratio of the product. The disadvantage of this method is that the synthesis process involves complex polymerization and crosslinking reaction, so it is difficult to modulate the pore size of the three-dimensional network [28]. Therefore, suitable commercial hydrogel has been chosen and used as a template combining with other synthetic strategies to control the synthesis of inorganic nanomaterials. Liu et al. [31] reported a method for preparing hydroxyapatite/dense hydrogel nanocomposites closely similar to the structure of bone. This method includes the promoting cations and anions diffusing respectively into the dense hydrogel templates under a direct current field, meeting, nucleating and growing process of hydroxyapatite nanoparticles. Their research not only contributes to the preparation of bone substitute materials, but also provides a new idea and method for preparing nanomaterials with hydrogel template. The disadvantage is that the concentration of hydrogels cannot be adjusted, and nor can the size of nanomaterials be controlled.

Agar hydrogels are easy to prepare without by-products and can be directly obtained by mixing agar with hot deionized water. The size of the three-dimensional network structure of the hydrogel can be controlled by changing the concentration of the agar hydrogel. In our previous work [35,36], a novel ion diffusion method controlled by ion exchange membrane was used for synthesizing RuO_2_·*n*H_2_O and Ni(OH)_2_ nanomaterials. In this paper, we combine ion diffusion method with agar hydrogel template method directly to synthesize Mn_3_O_4_ nanomaterials without adding any oxidizing agents. The Mn_3_O_4_ nanoparticles can directly nucleate and grow in the three-dimensional network structure of agar hydrogel and then the template is removed by calcination to obtain pure Mn_3_O_4_ nanoparticles. By changing the concentration of agar hydrogel and thus controlling the morphology and size of Mn_3_O_4_ nanoparticles, the electrochemical performance of Mn_3_O_4_ nanoparticles has been significantly improved.

## 2. Materials and Methods

### 2.1. Materials

Manganese sulfate (MnSO_4_) and sodium sulfate (Na_2_SO_4_) were purchased from Tianjin Fuchen Chemical Reagents (Tianjin, China), sodium hydroxide (NaOH) was purchased from Beijing Chemical Works (Beijing, China), and agar (biochemical reagent) was purchased from Tianjin Dingshengxin Chemical Industry Co. Ltd. (Tianjin, China). All reagents were analytical grade and used without further purification.

### 2.2. Synthesis

**Figure 1:**
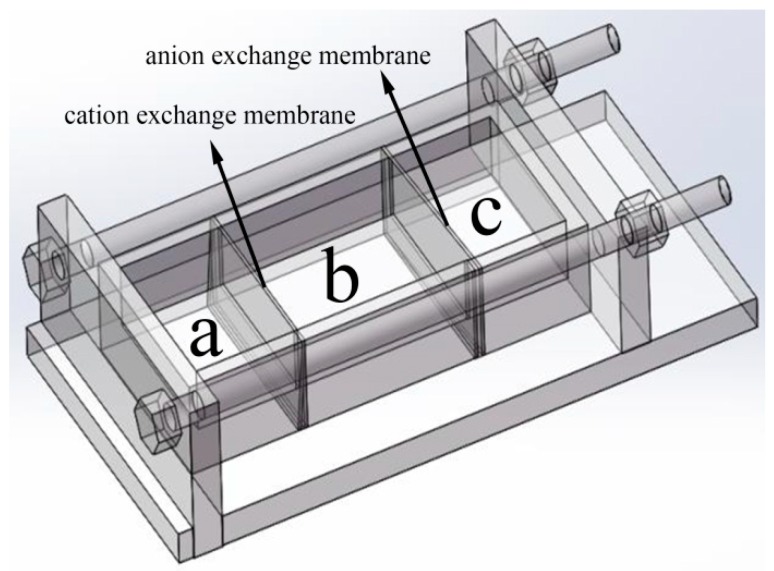
The ion diffusion reaction device controlled by ion exchange membrane.

The Mn_3_O_4_ nanoparticles were prepared with the reaction device reported in our previous work [35]. The reaction device is shown in Figure 1, the cation exchange membrane is fixed between the groove chamber a and b, and anion exchange membrane is fixed between groove chamber b and c. 50 mL of 0.5 mol L^−1^ Manganese sulfate was added into groove chamber a, and 50 mL of 0.6 mol L^−1^ Sodium hydroxide was added into groove chamber c. Then 100 mL different concentrations of agar hydrogel (1.0, 1.5, 2.0, 2.5 g L^−1^) were respectively added into groove chamber b. The reaction was carried out at 30 °C for 12 h. Due to the concentration difference, Mn^2+^ and OH^−^ diffused into groove chamber c through cation exchange membrane and anion exchange membrane, respectively. Mn^2+^ reacted with OH^−^ in the groove chamber c and a brown-black product was obtained by centrifugation. The resulting Mn_3_O_4_ was repeatedly rinsed with deionized water and ethanol and dried at 70 °C for 12 h, following by calcination at 350 °C for 2 h to obtain pure Mn_3_O_4_. Mn_3_O_4_ nanoparticles prepared with different concentrations of agar hydrogel were named M1.0, M1.5, M2.0 and M2.5, respectively. For example, M1.5 represents the sample prepared at 1.5 g L^−1^ of agar hydrogel solution. Mn_3_O_4_ sample was also prepared in the same manner without adding any hydrogel template agent and named M0.

### 2.3. Characterization

The phase structure and crystallinity of the samples were identified by X-ray diffraction (XRD, ULTIMAIV RIGAKU, Tokyo, Japan) in the range of 10–80° with a scan rate of 10°/min. The morphology of the samples was investigated using S-4800 field emission scanning electron microscopy (FESEM, Hitachi, Tokyo, Japan) and transmission electron microscopy (TEM, Tecnai G2 F20, Hillsborough, OR, USA). The valence states of Mn in the samples was characterized by X-ray photoelectron spectroscopy (XPS, PHI QUANTERA-II, ULVAC-PHI, INC., Tokyo, Japan) using amonochromatic Al K X-ray source (h = 1486.6 eV). The BELSORP-max specific surface area and pore size distribution instrument (ANKERSMID B.V. Holland, Nijverdal, Netherlands) were used to determine the pore size distribution and specific surface area.

### 2.4. Electrode Preparation and Electrochemical Characterization

80 wt % active materials (Mn_3_O_4_), 15 wt % acetylene black and 5 wt % polytetrafluorethlene (PTFE) were blended with a few drops of ethanol and then stirred to form well-mixed slurry to prepare working electrode. The resulting slurry was evenly coated on the current collector (nickel foam) with an area of 1 cm^2^ and dried at 70 °C for 12 h, and the mass of the whole material loading on nickel foam was about 8 mg. Finally, the electrode was pressed at 10 MPa. Cyclic voltammetry (CV), galvanostatic charge-discharge (GCD) and electrochemical impedance spectroscopy (EIS) tests were studied using CHI760E electrochemical workstation and 1 mol L^−1^ Na_2_SO_4_ aqueous solution was used as electrolyte. The CV and GCD analyses were performed in the potential window range of −0.2–0.8 V. EIS measurement was tested between 0.01 Hz and 100 kHz. The electrode test was carried out in a three-electrode system with nickel foam coated with Mn_3_O_4_ nanomaterials as the working electrode, saturated calomel electrode (SCE) as the reference electrode, and platinum foil as the counter electrode.

## 3. Results and Discussion

### 3.1. Morphology and Structure

**Figure 2:**
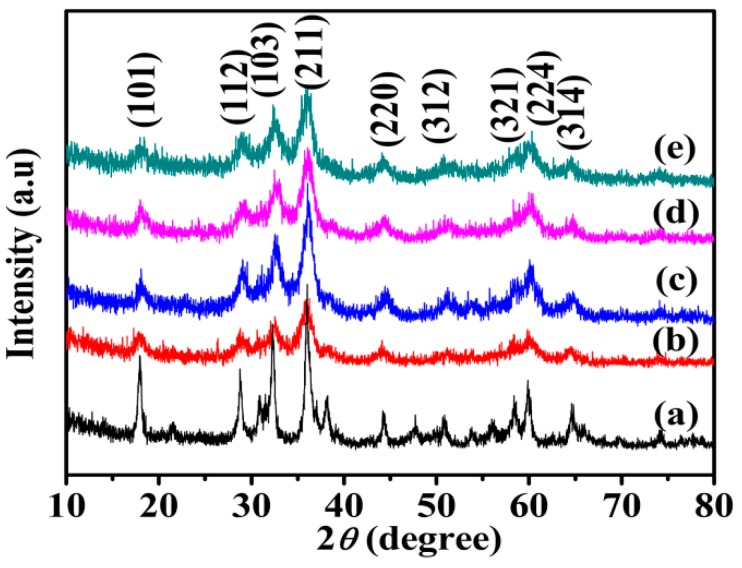
X-ray diffraction (XRD) patterns of Mn_3_O_4_ samples synthesized with different concentrations of agar hydrogel. (a) M0, (b) M1.0, (c) M1.5, (d) M2.0, (e) M2.5.

Figure 2 shows XRD pattern of Mn_3_O_4_ samples. The diffraction peaks at 18.0°, 29.0°, 32.5°, 36.2°, 44.6°, 53.9°, 58.8°, 60.0° and 64.7° correspond to (101), (112), (103), (211), (220), (312), (321), (224) and (314) planes of Mn_3_O_4_, respectively. All the diffraction peaks are indexed to the tetragonal hausmannite Mn_3_O_4_ (JCPDS 01-1127). No other impurity peaks appear, which reveals that pure hausmannite is obtained [37]. The diffraction peaks of Mn_3_O_4_ crystals become weaker and wider obviously in the existence of agar hydrogel template agent, indicating lower crystallinity or smaller size of Mn_3_O_4_.

**Figure 3:**
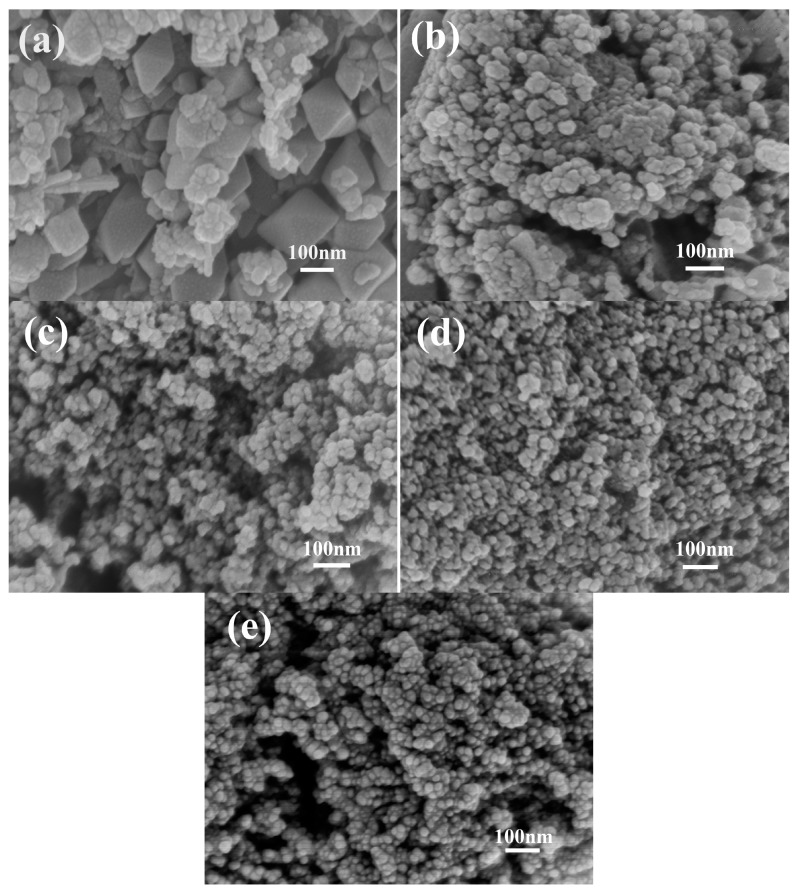
Scanning electron microscopy (SEM) images of Mn_3_O_4_ samples synthesized with different concentrations of agar hydrogel. (**a**) M0, (**b**) M1.0, (**c**) M1.5, (**d**) M2.0, (**e**) M2.5.

Figure 3a shows that M0 is constructed from irregularly tetragonal bipyramids and flaky particles with size of 30–150 nm. With increasing concentration of agar hydrogel (Figure 3b–d), the morphology of Mn_3_O_4_ nanoparticles gradually becomes irregularly spherical shape, the size of Mn_3_O_4_ nanoparticles becomes smaller and more uniform. M2.5 (Figure 3e) shows more regularly spherical shape and the minimal size of 10–20 nm. When the concentration of agar hydrogel is lower (M1.0 and M1.5), the aggregation behavior of Mn_3_O_4_ nanoparticles is improved. However, with the increase of concentration of agar hydrogel (M2.0 and M2.5), the structure of Mn_3_O_4_ nanoparticles becomes more compact. As shown in Figure 3c, M1.5 looks looser and exhibits irregularly spherical particles with the size of about 20–30 nm and the existence of abundant mesopores, which is advantageous for the full contact between electrolyte and active materials, and the enhancement of the capacitance.

**Figure 4:**
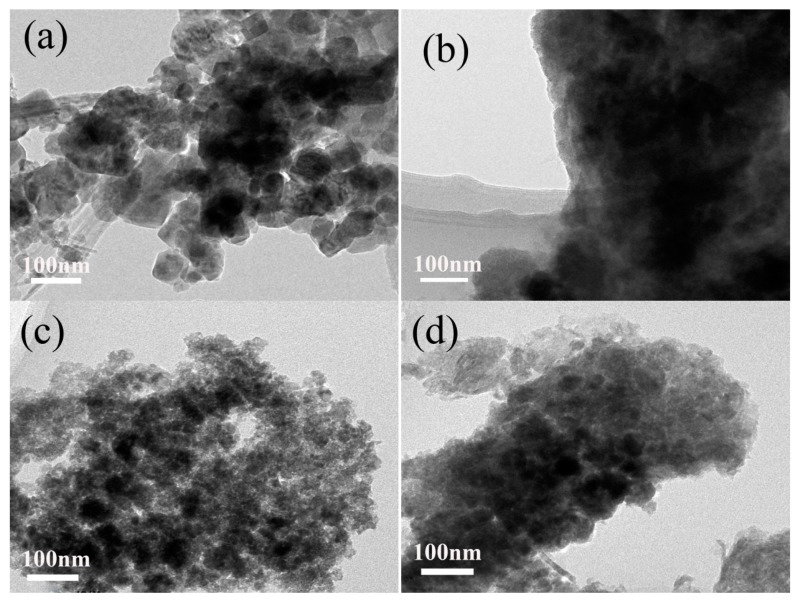
Transmission electron microscopy (TEM) images of Mn_3_O_4_ samples. (**a**) M0, (**b**) M1.5 (before calcination), (**c**) M1.5, (**d**) M2.5.

Figure 4 shows the TEM images of Mn_3_O_4_ nanomaterials synthesized with different concentrations of agar hydrogel, it is found that the agar hydrogel could effectively change the size and morphology of Mn_3_O_4_ nanomaterials. M0 (Figure 4a) shows the irregularly tetragonal bipyramids and flaky particles with size of 30–150 nm assembled by finer Mn_3_O_4_ nanoparticles. Figure 4b shows the TEM image of M1.5 before calcination, the Mn_3_O_4_ nanoparticles are covered with agar hydrogel template, and cannot be observed clearly. After the agar hydrogel template is removed by calcination, M1.5 (Figure 4c) shows that the irregularly spherical particles are constructed from smaller primary particles with size of about 5 nm, and there are many mesopores of different sizes in M1.5. When increasing the concentration of agar hydrogel, the morphology of M2.5 becomes more compact. The results of TEM are consistent with those of SEM and the enhancement of the capacitance.

The SEM and TEM results suggest that agar hydrogel with three-dimensional network plays an important role in the size and shape-controlled synthesis process of Mn_3_O_4_ nanoparticles. The suitable concentration of agar hydrogel can effectively regulate the morphology, particle size and distribution of Mn_3_O_4_, and form more mesopores.

**Figure 5:**
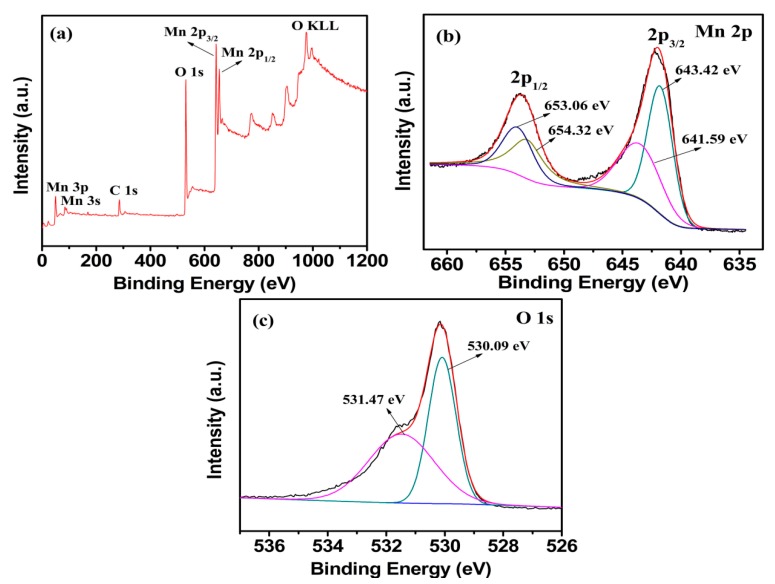
X-ray photoelectron spectroscopy (XPS) spectra of the (**a**) survey scan, (**b**) Mn 2p, (**c**) O 1s electron XPS spectra for M1.5.

Figure 5a shows the XPS survey scan analysis of M1.5 in the binding energy range 0–1200 eV. Except for the contaminant carbon, no significant impurities are founded. As shown in Figure 5b, the Mn 2p_3/2_ spectrum shows two distinct peaks at 641.59 and 643.42 eV, which are consistent with the binding energy of Mn(II) to Mn(III) in Mn^2+^(Mn^3+^)_2_O_4_ [27]. The O 1s peak of Mn_3_O_4_ splits into two peaks with the binding energy 530.09 and 531.47 eV in Figure 5c, which are in agreement with the analysis of O 1s in Mn_3_O_4_ [38]. All the results prove that pure Mn_3_O_4_ is obtained, which is consistent with the XRD analysis. The mole ratio of total manganese to the oxygen (Mn/O) calculated from Figure 5b,c is 1.54, which is slightly higher than the theoretical value (1.33). It is presumed that the excess oxygen is due to the residual oxygen produced by incomplete carbonization of agar hydrogel.

**Figure 6:**
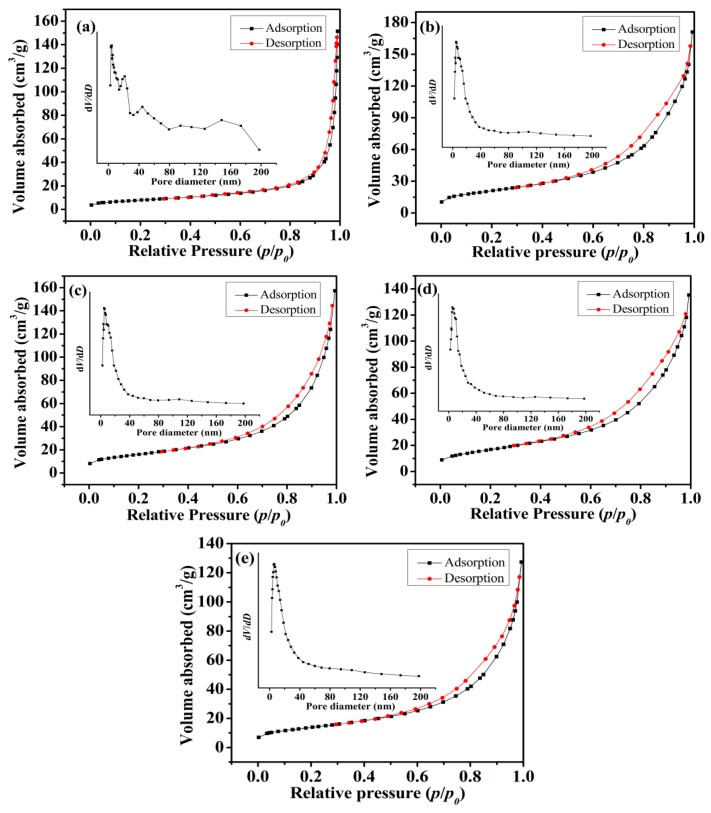
Nitrogen adsorption-desorption isotherms and the pore size distribution (insert) of Mn_3_O_4_ samples. (**a**) M0, (**b**) M1.0, (**c**) M1.5, (**d**) M2.0, (**e**) M2.5.

The BET surface area and pore size distributions of as-prepared Mn_3_O_4_ particles synthesized at different concentrations of agar hydrogel were investigated by N_2_ adsorption-desorption measurement. As shown in Figure 6, according to the International Union of Pure and Applied Chemistry (IUPAC) classification of adsorption and desorption isotherms, all five isotherm profiles can be classed as type IV with a hysteresis loop in the relative pressure range of 0.55–0.75, which indicate that the samples have the adsorption properties of porous materials [39]. For M1.0, M1.5, M2.0 and M2.5 samples, the hysteresis loop shifts to lower relative pressure, indicating smaller pores size. The Barrett-Joyner-Halenda (BJH) pore size distribution curves demonstrate the existence of more abundant mesopores and relatively homogeneous pore distribution for the Mn_3_O_4_ samples synthesized using agar hydrogel template. Table 1 exhibits the results of the measured BET specific surface area and the BJH pore size distribution of the samples, the entire samples prepared using agar hydrogel template (M1.0, M1.5, M2.0 and M2.5) have much higher specific area than that prepared without using any template (M0). For M1.0, it is suggested that the largest specific area of 75.7 m^2^ g^−1^ comes from the more micropores and smaller mesopores formed by the assembly of finer Mn_3_O_4_ nanoparticles, the micropores and smaller mesopores are disadvantageous to the diffusion of electrolytes. M2.5 has the smallest specific surface area of 49.6 m^2^ g^−1^, due to the close packing of Mn_3_O_4_ nanoparticles. M1.5 has the largest average pore diameter of 15.9 nm, higher specific area and larger pore volume resulting from the abundant and larger mesopores (Figure 6c inset). These results are consistent with that of SEM and TEM.

### 3.2. Electrochemical Performance of Mn_3_O_4_ Electrode

**Figure 7:**
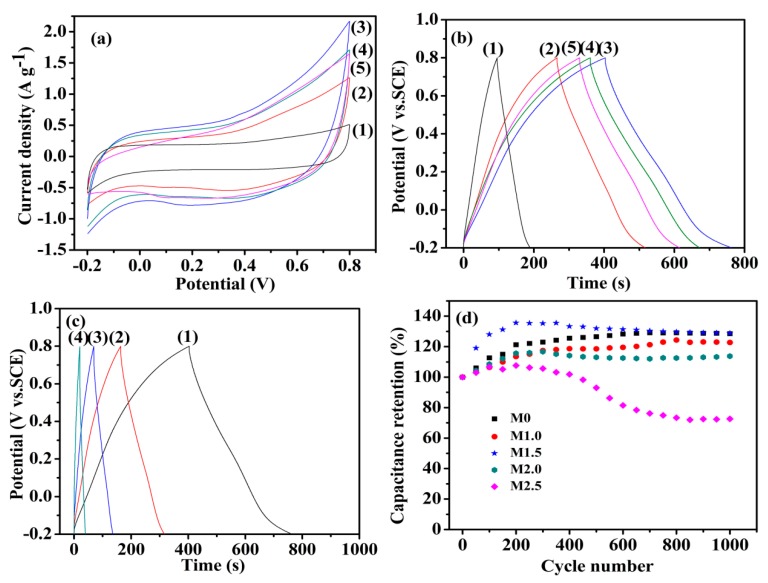
Cyclic voltammetry (CV) curves (**a**) at 5 mV s^−1^ and galvanostatic charge-discharge (GCD) curves (**b**) at 0.5 A g^−1^ (1) M0, (2) M1.0, (3) M1.5, (4) M2.0, (5) M2.5; (**c**) Galvanostatic charge-discharge curves of M1.5 electrode at (1) 0.5 A g^−1^, (2)1.0 A g^−1^, (3) 2.0 A g^−1^, (4) 5.0 A g^−1^; (**d**) The relation between capacitance retention and the charge-discharge cycle times of Mn_3_O_4_ samples.

In order to investigate the capacitive performance of the as-prepared Mn_3_O_4_, CV and GCD tests were employed. Figure 7a shows the CV curves of Mn_3_O_4_ nanoparticles synthesized with different concentrations of agar hydrogel at the scan rate of 5 mV s^−1^. All the CV curves of the as-prepared materials exhibit a symmetrical quasi-rectangular shape, which reveals the pseudocapacitive behaviors and good reversibility [40,41]. The pseudocapacity of manganese oxide is attributed to the redox exchange of protons or cations in the electrolyte as the following equation:

MnO*_a_*(OH)*_b_* + *n*H^+^*n*e^−^ ⇔ MnO*_a_*_−_*_n_*(OH)*_b_*_+_*_n_* (1)

where, MnO*_a_*(OH)*_b_* and MnO*_a−n_*(OH)*_b_*_+*n*_ represent the high oxidation state and low oxidation state of manganese, respectively. The electrochemical performance of the electrode materials is positively related to the area enclosed by the CV curves. Therefore, it can be concluded that M1.5 has the largest specific capacitance from Figure 7a. The specific capacitances of the Mn_3_O_4_ prepared using agar hydrogel template agent were higher than that of M0. The higher specific capacitance of Mn_3_O_4_ synthesized with agar hydrogel template result from the higher specific area and the presence of more mesopores. The higher specific surface area can increase the contact area between the electrode active materials and electrolyte, and the more charges can be transferred into the inside of electrode material through the electrochemical reaction at the interface, which makes the active materials to be fully reacted and thus greatly improving the electrochemical properties [42,43].

The GCD curves of Mn_3_O_4_ electrodes at the current density 0.5 A g^−1^ are shown in Figure 7b, the specific capacitance of Mn_3_O_4_ electrode can be calculated from the GCD curves according to the following equation:

(2) $$C\;=\;\frac{I\;\times \;\Delta t}{m\;\times \;\Delta E}$$

where *C* (F g^−1^) is the specific capacitance; *I* (A) is the discharge current; △*t* (s) is the discharge time; *m* (g) is the mass of active material; △*E* (V) is the GCD potential range. According to the discharge curves, it can be calculated that the specific capacitance values of M0, M1.0, M1.5, M2.0 and M2.5 are 46.5, 126.1, 183.0, 156.8 and 143.3 F g^−1^ at the current density 0.5 A g^−1^, respectively. The specific capacitance of M1.5 is higher than that of Mn_3_O_4_ nanomaterials reported in the literature [25,26]. It is suggested that the highest specific capacitance of M1.5 is due to the fact that M1.5 has more abundant mesopores, which will promote the diffusion of electrolyte ions into the interior of electrode active materials [27,44].

Figure 7c shows the GCD curves of M1.5 electrode at different current densities. The charging curves and discharging curves are approximately symmetrical, indicating that the electrode active material is relatively stable and highly reversible. The specific capacitance of M1.5 evaluated from the discharge curves are 183.0, 154.7, 134.8, 102.0 F g^−1^ at current densities of 0.5, 1.0, 2.0, 5.0 A g^−1^, respectively. When the current density is low, the Mn_3_O_4_ electrode materials can be sufficiently and effectively wetted by the electrolyte and more active sites of electrode materials are exposed to the electrolyte, which can increase the utilization of active materials and make them participate more fully in the redox reaction [37,44]. The specific capacitance shows a decreasing trend with the current densities increase, which is due to the gradually reduction of protons entering to the inside of active materials, and thus causing a transfer of redox reactions gradually from the inside of Mn_3_O_4_ nanoparticles to the surface of electrode [45,46]. The cyclic stability of Mn_3_O_4_ electrodes were employed in charge-discharge test at 2.0 A g^−1^ up to 1000 cycles. As shown in Figure 7d, all the Mn_3_O_4_ electrodes have an outstanding cycling performance except M2.5. The specific capacitance retention of Mn_3_O_4_ electrodes increases gradually and reaches a highest value, and thereafter slowly decreases, the phenomenon may be attributed to the following reasons. First, electrolyte ions gradually penetrate into the interior of electrode materials and open more pore of electrode materials at the beginning stage of charge-discharge cycles, which will enhance the faradic reactions [47]. Second, the electrode is gradually wetted, which will increase the active sites of electrode materials and promote the faraday reaction [48,49]. Third, the abundant mesoporous provide good buffer volume expansion capability and increase the cyclic stability of the electrodes [50]. Due to the smaller pore size of M2.5, the structure of M2.5 will be changed and thus formed a dense inner layer after repeated charge-discharge test, which is not conductive to the electrode reactions and the flowing of electrolyte, and lead to a rapid decrease of the capacitance retention [44]. However, the samples synthesized under lower agar hydrogel concentration maintain the same high capacitance retention as M0, which means that suitable concentration of agar hydrogel template will not influence the stability of Mn_3_O_4_ electrode.

**Figure 8:**
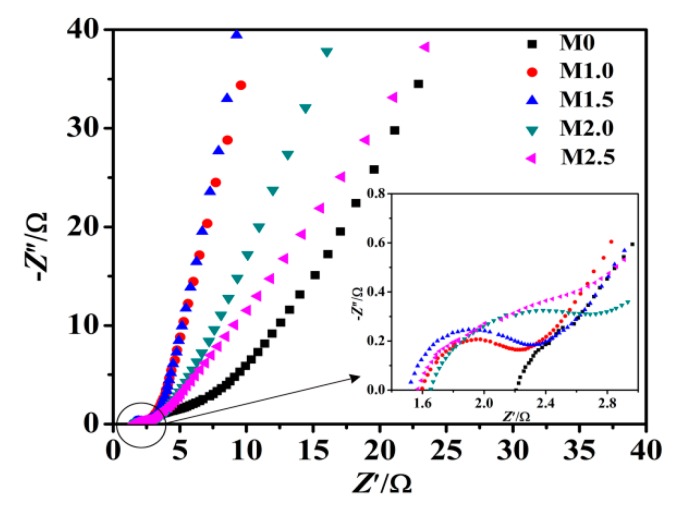
Nyquist plots of as-prepared samples electrodes.

EIS measurements were performed to further study the interfacial ion diffusion and charge transfer process of the Mn_3_O_4_ electrodes. As shown in Figure 8, the Nyquist plot of Mn_3_O_4_ electrodes is consisted of the approximate semicircle in high-frequency region and a straight line with certain slop in low-frequency region, which is consistent with the characteristics of pseudocapacitor. The electrode process is controlled by electrochemical polarization in high-frequency region, due to the internal resistance (*R*s) and charge-transfer resistance (*R*ct) of the active materials redox reaction [44,51]. The intercept of the Nyquist plot in the real axis represents the *R*s value, and the semicircle radius represents the *R*ct value. According to the Figure 8, the *R*s values of M0, M1.0, M1.5, M2.0 and M2.5 are 2.205, 1.595, 1.507, 1.653 and 1.568 Ω cm^2^, respectively. The *R*ct values of M0, M1.0, M1.5, M2.0 and M2.5 are 1.794, 0.575, 0.708, 0.715 and 0.714 Ω cm^2^, respectively. The *R*s and *R*ct value of M0 is the largest, and *R*s value of M1.5 is the smallest, indicating that Mn_3_O_4_ with higher specific surface area and more abundant mesopores is advantageous to reduce the charge transfer resistance of the electrode/electrolyte interface [52]. In the low-frequency region, the electrode impedance plot is a straight line, showing that the electrolytes diffusion is the determining step of electrode process [53], which indicates the diffusion resistance (Warburg resistance *Z*w) of electrolyte into active materials and the level of proton transfer, the larger liner slope in the low-frequency region indicating the smaller *Z*w value, higher transfer rate of protons and electrolyte ions. The straight lines of M1.0, M1.5 and M2.0 at low frequency are much steeper than that of M0, indicating M0 has the larger *Z*w compared with M1.0, M1.5 and M2.0. M1.5 has the highest conductivity, which are advantageous for the improvement of chemical performance.

In a word, the synthetic strategy used in this work can control the morphology and particle size of the as-synthesized Mn_3_O_4_ nanoparticles by changing the concentration of agar hydrogel template, and endow the Mn_3_O_4_ nanoparticles with higher specific surface area and abundant mesoporous, which is conducive to improving the electrochemical performance. It should be pointed out that this part of the work is the first attempt to synthesize Mn_3_O_4_ nanoparticles using this novel synthetic strategy, and we will continue the synthesis of other inorganic nanomaterials, with a view to further improving this method and expanding its application field.

## 4. Conclusions

The irregularly spherical Mn_3_O_4_ nanoparticles were successfully synthesized via an ion diffusion method controlled by ion exchange membrane combining with agar hydrogel template without any oxidizing agents. It is discovered that the structure and electrochemical behavior of Mn_3_O_4_ nanoparticles are closely dependent on the concentration of agar hydrogel. The SEM and TEM analysis reveal that agar hydrogel template could adjust the morphology and size of Mn_3_O_4_ nanoparticles. BET analysis indicates that Mn_3_O_4_ synthesized with hydrogel template all have much higher specific area and abundant mesoporous than that synthesized without hydrogel template. Mn_3_O_4_ nanoparticles can directly nucleate and grow in the three-dimensional network structure of agar hydrogel, which can reduce the agglomeration of Mn_3_O_4_ nanoparticles and thus increase its specific area. Mn_3_O_4_ sample synthesized with 1.5 g L^−1^ of agar hydrogel solution (M1.5) has the highest specific capacitance of 183.0 F g^−1^ in 1.0 mol L^−1^ Na_2_SO_4_ electrolyte at the current density of 0.5 A g^−1^, which is increased by 293% compared with that of Mn_3_O_4_ synthesized without any template agent. The charge-discharge tests show that suitable concentration of agar hydrogel template will not influence the stability of Mn_3_O_4_ electrode.

The as-prepared Mn_3_O_4_ nanoparticles show high electrochemical performance, which is ascribed to the improvement of the conductivity and utilization of Mn_3_O_4_ nanomaterials. The ion diffusion method controlled by ion exchange membrane combining with agar hydrogel template is a novel approach for preparing inorganic nanomaterials, which has the advantages of mild reaction conditions, easy operation, energy saving and environmental friendliness.

## Figures and Tables

**Table 1:** BET surface area and pore volume of as-prepared Mn_3_O_4_.

Samples	Surface Area, *S*_BET_ (m^2^ g^−1^)	Average Pore Diameter, AP (nm)	Total Pore Volume, *V*_tot_ (cm^3^ g^−1^)
M0	28.64	32.24	0.231
M1.0	75.70	13.56	0.257
M1.5	58.79	15.91	0.234
M2.0	62.64	12.89	0.202
M2.5	49.61	15.31	0.190
